# Axillary Artery Variant: A Cadaveric Case Report and Clinical Significance

**DOI:** 10.7759/cureus.91533

**Published:** 2025-09-03

**Authors:** Austin Varghese, Kaitlyn Rowland, Lauren Ellingham, Ivan Rubalcava, Grete Hamic, Scott Kilian, Roni Wu, Sama Jwaied, Arunabh Bhattacharya

**Affiliations:** 1 Applied Biomedical Sciences, University of the Incarnate Word School of Osteopathic Medicine, San Antonio, USA; 2 Applied Biomedical Sciences, University of Incarnate Word, San Antonio, USA

**Keywords:** axillary artery bifurcation, brachial plexus, deep axillary branch, superficial axillary branch, thoracic outlet syndrome

## Abstract

Vasculature variations are commonly studied and used as a tool to further understand embryology, pathology, and clinical significance. In this case report, a rare axillary artery variant (AA) in the right upper limb of an 84-year-old Caucasian male donor was analyzed (including its relationship with the brachial plexus) and differentiated from the canonical AA branching pattern seen in the left upper limb. The variant was observed as a bifurcation of the second part of the AA into the superficial axillary branch (SAB) and deep axillary branch (DAB). The SAB gave off the lateral thoracic artery and a muscular branch providing vasculature to the serratus anterior (SA) muscle before continuing distally as the brachial artery. The DAB traveled deep to the lateral cord’s contributing root to the median nerve and directly gave rise to the thoracodorsal, anterior circumflex humeral, posterior circumflex humeral, and circumflex scapular arteries before continuing posteriorly as the profunda brachii artery. The subscapular artery was absent. In addition to its normal blood supply to the latissimus dorsi muscle, the thoracodorsal artery provided branches to the SA and teres major muscles. These findings not only advance the scientific knowledge of the AA as a highly variable vessel but also enrich the clinical training of practitioners focused on accurate diagnosis of the causes of neuropathies of the upper limb and on improving patient outcomes in surgical or interventional procedures.

## Introduction

**Figure 1:**
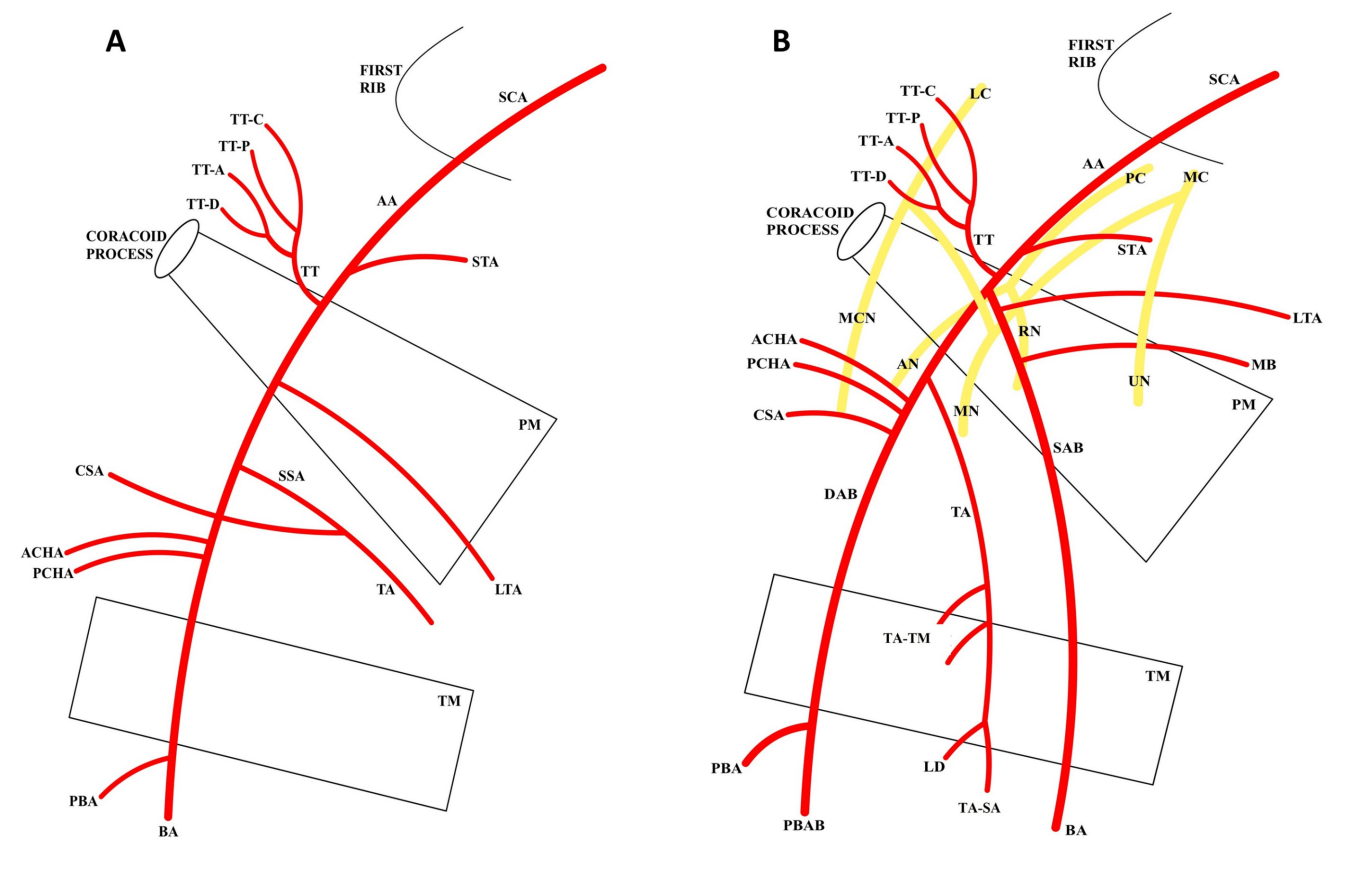
Diagrams illustrating (A) canonical and (B) variant axillary artery anatomy. The relationship of the variant to the brachial plexus is demonstrated in B. SCA: subclavian artery, AA: axillary artery, SAB: superficial axillary branch, DAB: deep axillary branch, STA: superior thoracic artery, TT: thoracoacromial trunk, LTA: lateral thoracic artery, SSA: subscapular artery, TA: thoracodorsal artery, CSA: circumflex scapular artery, ACHA: anterior circumflex humeral artery, PCHA: posterior circumflex humeral artery, BA: brachial artery, PBA: profunda brachii artery, PBAB: PBA branch to long head of triceps brachii muscle, TA-TM: TA branch to teres major muscle, TA-SA: TA branch to serratus anterior muscle; MB: muscular branch to serratus anterior muscle, TT-C: clavicular branch of TT, TT-P: pectoral branch of TT, TT-A: acromial branch of TT, TT-D: deltoid branch of TT, LC: lateral cord, MC: medial cord, PC: posterior cord, UN: ulnar nerve, RN: radial nerve, MN: median nerve, AN: axillary nerve, MCN: musculocutaneous nerve, PM: pectoralis minor muscle, TM: teres major muscle, LD: latissimus dorsi muscle.

The axillary artery (AA) originates at the lateral border of the first rib and terminates at the inferior border of the teres major (TM) muscle to become the brachial artery (BA). The AA courses anterior to the posterior cord of the brachial plexus (BP) and is divided into three parts in relation to the pectoralis minor (Pm) muscle. The first part ends at the proximal aspect of the Pm muscle and gives rise to only one artery, the superior thoracic artery (STA), which supplies the superior part of the serratus anterior (SA) muscle and the first two intercostal spaces [1]. The second part of the AA lies deep to the Pm muscle and gives rise to two arteries: the thoracoacromial trunk (TT) and the lateral thoracic artery (LTA). The TT splits into four branches deep to the clavicular head of the pectoralis major (PM) muscle to provide vasculature to the sternoclavicular joint, the subclavius, PM, Pm, and deltoid (DE) muscles, and the breasts. The LTA travels inferiorly along the lateral border of the Pm muscle and provides blood supply to the SA muscle and the lateral aspect of the breast. The third part of the AA lies distal to the Pm muscle and proximal to the inferior border of the TM muscle. Three arteries originate here: the anterior and posterior circumflex humeral arteries (ACHA, PCHA) and the subscapular artery (SSA) (Figure 1A). The circumflex humeral arteries anastomose around the surgical neck of the humerus. In addition, they provide blood supply to the TM, teres minor (Tm), and DE muscles. The SSA travels inferiorly along the lateral border of the subscapularis muscle on the posterior axillary wall and divides into the circumflex scapular artery (CSA) and thoracodorsal artery (TA) (Figure 1A). The CSA provides blood supply to the DE, Tm, and triceps brachii (TB) muscles, the glenohumeral joint, and the cutaneous region over the scapula. The TA supplies the latissimus dorsi (LD), intercostal and SA muscles, and the skin of the lateral surface of the thoracic wall [1].

AA variations are relatively common and often explained by dysregulation in embryological development [2,3]. Population-based cadaveric studies have observed a canonical AA branching pattern in only 10-40% of the upper limbs, further suggesting that AA variations are more frequent than the canonical branching distribution of the AA [3,4]. Most of these variations are unique vessel distributions either in the second or third part of the AA, such as an absent TT with its four branches originating from the AA, a common trunk for the ACHA, PCHA, SSA, and profunda brachii artery (PBA), the LTA arising from either the SSA or TA, or the PCHA arising from the SSA [3,4]. Understanding possible AA anomalies can potentially increase the accuracy of diagnoses such as thoracic outlet syndrome (TOS) [5,6]. This knowledge may also improve patient outcomes postoperatively for procedures such as total mastectomies, axillary lymph node biopsy and dissection, surgical treatment of AA aneurysms, surgical treatment of TOS, and repair of trauma to the axilla, including fracture of the surgical neck of the humerus [6-9]. In this case report, we describe a rare bifurcation of the second part of the AA into superficial and deep branches, analyze its relationship with the brachial plexus, and discuss the potential clinical implications.

## Case presentation

**Figure 2:**
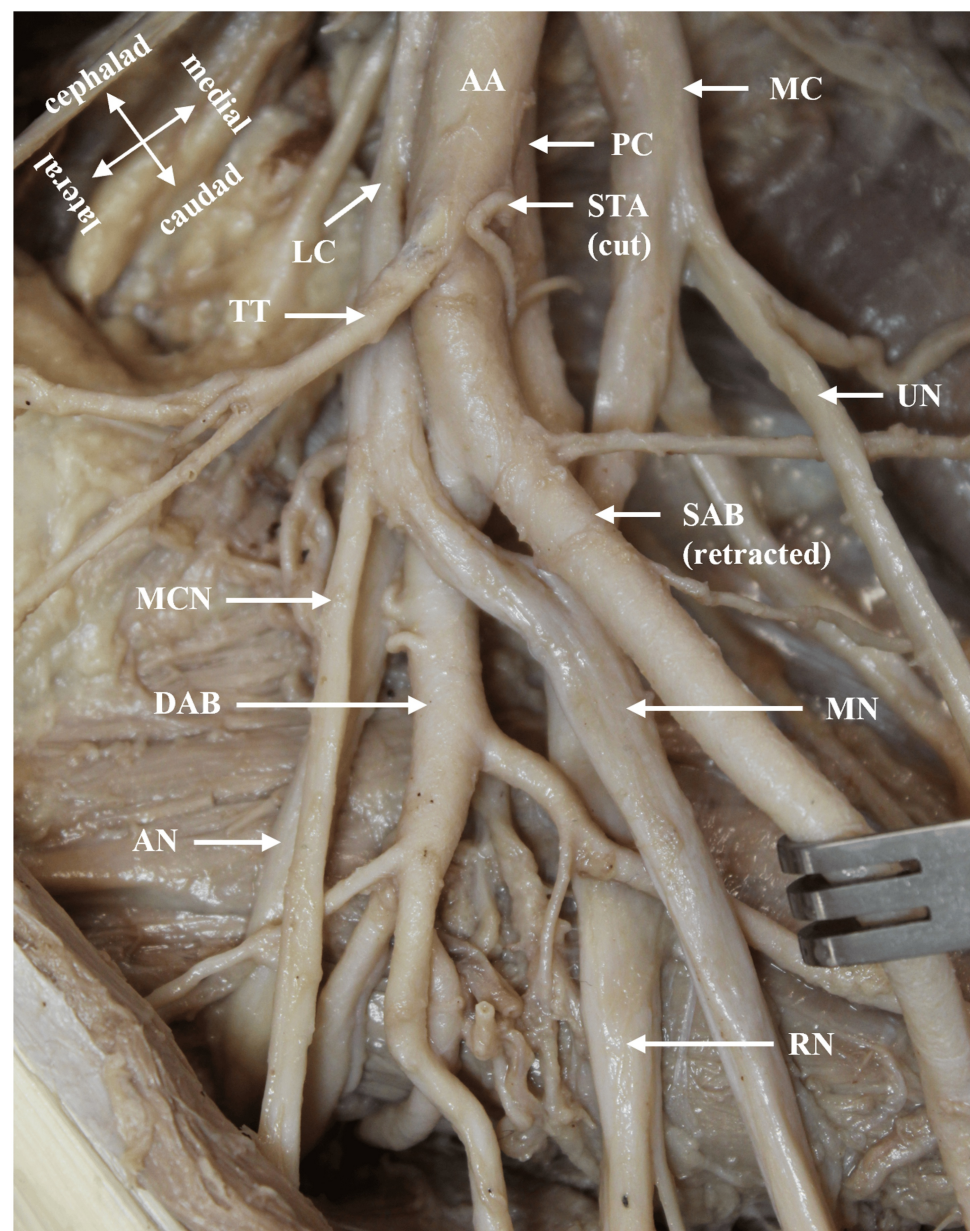
Anterior view of right axilla demonstrating the bifurcation of the AA. The pectoralis major and minor muscles are reflected and the arm abducted. The second part of the AA divides into SAB and DAB around the cords and terminating branches of the brachial plexus. AA: axillary artery; AN: axillary nerve; DAB: deep axillary branch; LC: lateral cord; MC: medial cord; MCN: musculocutaneous nerve; MN: median nerve; PC: posterior cord; RN: radial nerve; SAB: superficial axillary branch; STA: superior thoracic artery; TT: thoracoacromial trunk; UN: ulnar nerve.

**Figure 3:**
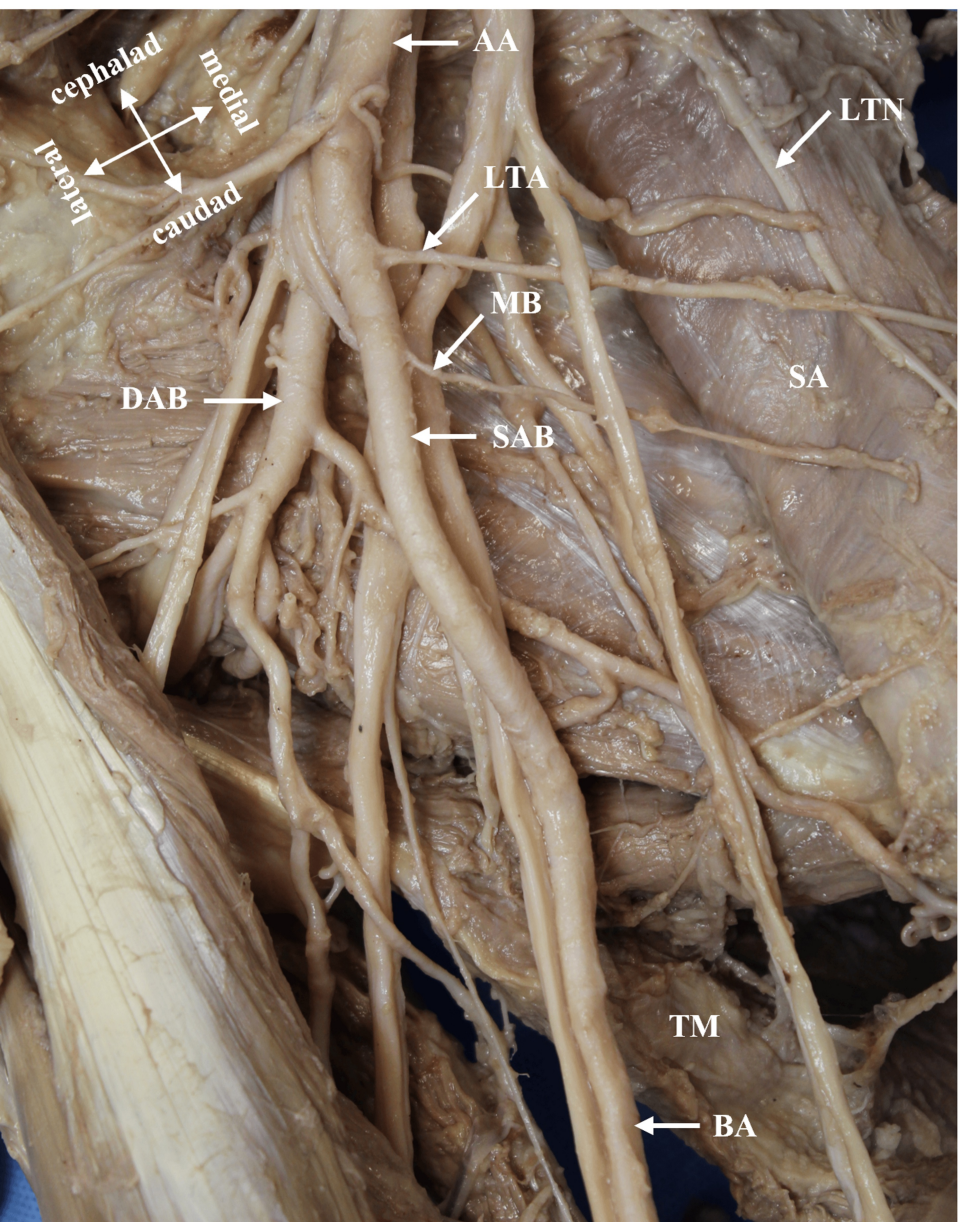
Anterior view of the right axilla and lateral thoracic wall demonstrating the branches of the SAB to the SA muscle. The pectoralis major and minor muscles are reflected and the arm abducted. The SAB gave rise to LTA and a MB to the SA muscle before continuing as the BA at the inferior border of the TM muscle. AA: axillary artery; BA: brachial artery; DAB: deep axillary branch; LTA: lateral thoracic artery; MB: muscular branch to the SA; LTN: long thoracic nerve; SA: serratus anterior muscle; SAB: superficial axillary branch; TM: teres major muscle.

**Figure 4:**
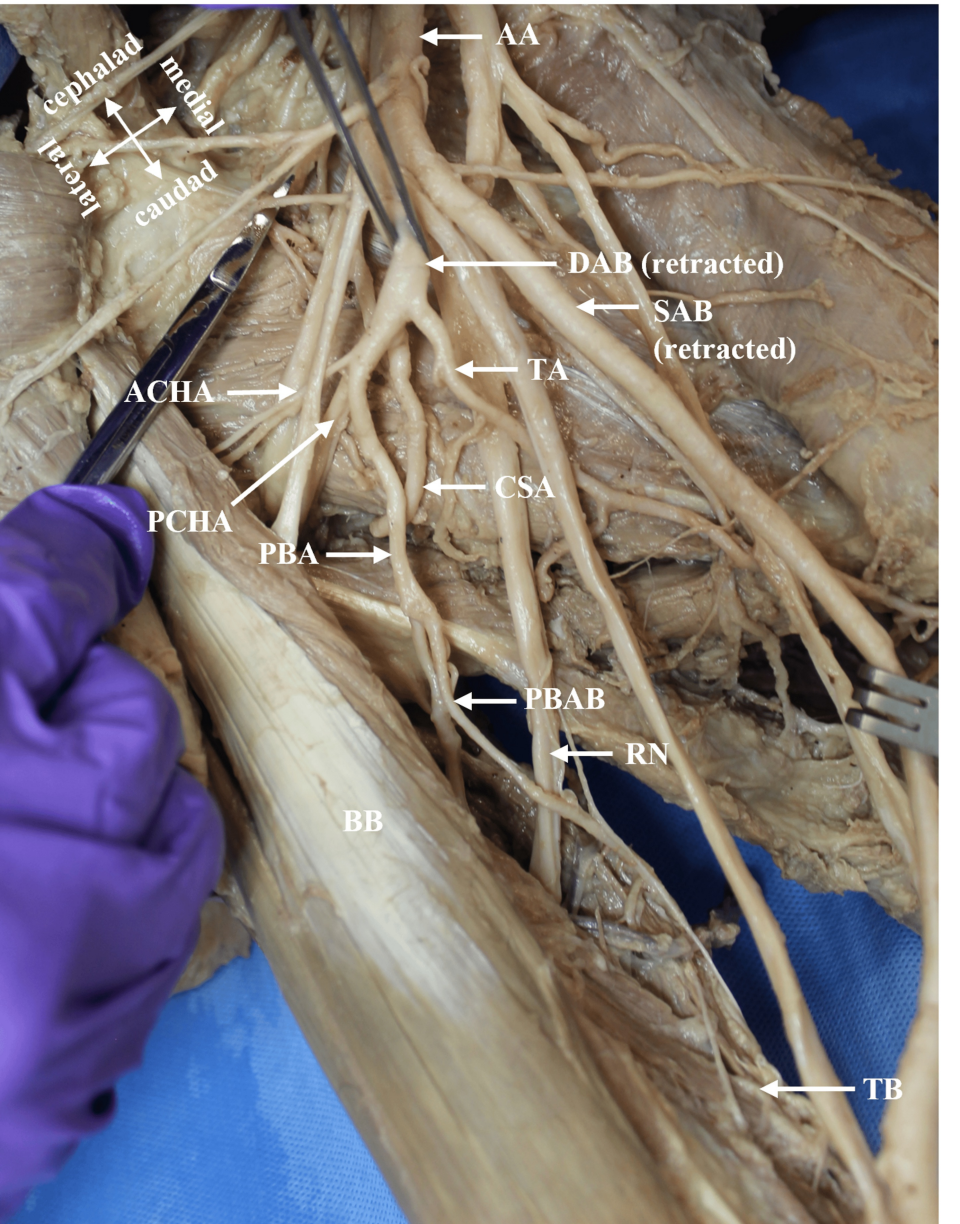
The ACHA, PCHA, CSA, and TA emerged as direct branches from the DAB. AA: axillary artery; ACHA: anterior circumflex humeral artery; BB: biceps brachii muscle; CSA: circumflex scapular artery; DAB: deep axillary branch; PBA: profunda brachii artery; PBAB: branch of PBA to the TB muscle; PCHA: posterior circumflex humeral artery; RN: radial nerve; SAB: superficial axillary branch; TA: thoracodorsal artery; TB: long head of triceps brachii muscle.

During a routine bilateral dissection of the upper limbs, an unusual branching pattern of the AA in the donor body was noted (race: Caucasian, age: 84 years, gender: male). The donor was obtained through the Willed Body Program at the University of Texas Health at San Antonio for the purposes of medical education and research. The AA had a canonical branching pattern on the left side of the axilla (Figure 1A). The variant was identified unilaterally in the right axilla in the second part of the AA, deep to the Pm muscle. The variant AA bifurcated into a superficial axillary branch (SAB) and a deep axillary branch (DAB) (Figures 1B, 2). The SAB coursed anterior to the medial cord’s (MC) contributing root to the median nerve (MN) and transitioned to the BA at the inferior border of the TM muscle (Figures 1B, 3). The SAB had a 4 mm diameter and gave off two branches that supplied the SA muscle: a proximal LTA and a distal muscular branch (Figures 1B, 3). The DAB measured 5 mm in diameter and traveled deep to the lateral cord’s (LC) contributing root to the MN (Figure 1B, 2). The ACHA, PCHA, CSA, and TA all originated directly from the DAB. The SSA was absent. In addition to its normal blood supply to the LD muscle, the TA gave off branches that supplied the SA and TM muscles. The DAB continued posteriorly as the PBA to enter the radial sulcus with the radial nerve. Before entering the radial sulcus, the PBA provided a branch that supplied the long head of the TB muscle (Figure 1B, 4).

## Discussion

Across the literature, variation in AA occurs quite commonly [3,4,10-12]. In a population study, Astik and Dave observed the canonical AA branching pattern in 30/80 limbs of Asian origin (37.5%) [4]. The remaining 62.5% of the limbs had a variation in the AA, either in its second or its third part [4]. In a recent population study in Caucasians (62 limbs), Thiele et al. reported that less than 20% of the limbs had canonical AA branching patterns [3]. The study found the LTA to originate from the TA in 37% of the limbs and from the SSA in 21% of the limbs [3]. In 13% of the limbs, the PCHA and ACHA emerged from a common trunk [3]. Additionally, the PCHA branched from the SSA in 19.4% of the limbs [3]. A recent scoping review reported AA variability to be highest in the third part, followed by the second part, with the first part being highly constant [9]. This case report presents a rare bifurcation at the second part of the AA around the MC and LC contributions to the MN. Similar to this case report, existing reports have documented the splitting of the AA at various parts of the artery [9,13-16]. Aastha et al. reported a branch from the third part of the AA, which they labeled as the common trunk [13]. This common trunk gave off the ACHA, PCHA, and SSA and continued as the PBA. The third part of the AA terminated as the BA in the arm [13]. Another study of 178 limbs (79 Caucasians, 10 Blacks) found only one of the limbs to have bifurcation of the AA in its second part (0.56%) [16]. The study labeled the bifurcating vessels as the brachial and deep brachial arteries, which is synonymous with the SAB and DAB in this case report. Variable origins of AA branches were also reported in the study [16]. For instance, the LTA and ACHA originated from the SSA, which branched from the BA. In addition, the TT originated from the BA instead of its canonical origin from the second part of the AA [16]. The study further reported the absence of the SSA in 1.7% of the cadavers [16], similar to what we found in our donor. The SSA is often viewed as a conserved branch found on the AA in 98.3% of cases [4]. However, a radiologic study by Barrett et al. that focused on analyzing the characteristics of the SSA and thoracodorsal arterial system found the SSA to be absent in 25/200 (12.5%) arterial systems [17]. Previously, a cadaveric population study by Xhakaza et al. that analyzed the origin of the SSA in 50 indigenous Black South Africans found the SSA to be absent in 11% (11/100) of their cadavers [18]. These findings suggest that an absent SSA might occur more frequently than is often stated in the literature.

The rare bifurcation of the AA at its second part and its distribution of vessels seen in our study offer valuable educational insight into the vessel’s vasculogenesis. These variations can be partly explained by disruptions in embryonic remodeling of the vascular plexus within the upper limb bud in utero. The AA and its branches on both sides of the human body originate from the axial artery, which is a continuation of the seventh intersegmental artery. Disruptions may arise during days 33-41 of AA development, AA penetration, and/or the formation of the BA, up until day 52 when the upper limb achieves its definitive shape and location [2,19].

A recent cadaveric case report identified a novel AA branch located at the third part of the AA, which entrapped the LC’s contributing root of the MN [14]. The distal axillary artery, which is the novel AA branch, and the common trunk branch were clamped onto the MN. In many ways, the common trunk branch compares to the SAB found in this case report and how it entraps the MN. The bifurcation of the second part of the AA around the MN could lead to a wide range of neurologic signs and symptoms, especially if dilation of the AA were to occur due to medication use, atherosclerosis, or arterial ectasia [8]. Entrapment of the MN due to this AA bifurcation could result in reduced or lost function and atrophy of the muscles innervated by it, in addition to paresthesia, hypoesthesia, or anesthesia in the MN distribution. Symptomatically, this impingement could present similarly to proximal MN injury in the upper arm, cubital fossa, or the BP. Weakness or loss of function in the muscles innervated by the MN and sensory abnormalities could also resemble the symptoms of carpal tunnel syndrome (CTS). However, this AA variation could additionally cause loss of sensation in the central palm, unlike CTS, because the compression of the MN occurs in the second part of the AA, as opposed to the wrist, where the carpal tunnel is located.

This variant could also present similarly to TOS with the entrapment of the second part of the AA beneath the Pm muscle. TOS occurs due to compression of the BP in the thoracic outlet, specifically between the clavicle and the first rib, between the anterior and middle scalene muscles, or beneath the Pm muscle [6]. TOS has similar symptoms to CTS but can also include pain in the neck and shoulder depending on the location of BP compression [6]. In this variant, with compression potentially occurring in the second part of the AA beneath the Pm muscle, a patient would likely present with an additional sign of a positive Wright’s test on physical examination, which causes reproduction of symptoms like those previously stated and a decrease in the radial pulse when the shoulder is abducted and externally rotated with the head turned away from the affected side [20]. Due to the extensive differential diagnoses possible for the signs and symptoms likely attributable to a similar AA variant, it is important for physicians to thoroughly investigate the area of MN entrapment before invasive intervention such as surgery.

Knowledge and understanding of AA variations are paramount for surgical procedures in the axillary region such as the dissection of lymph nodes in breast cancer patients. In addition, branches of the AA are frequently utilized for grafting in coronary bypass surgery and other cardiovascular operations due to their larger diameter and reduced involvement in atherosclerosis [8]. For instance, percutaneous AA access is a viable alternative to the commonly used transfemoral and transapical transcatheter aortic valve implantation [21]. This procedure is used primarily for severe symptomatic aortic stenosis in high-risk patients and those who are considered inoperable [21]. Current interventional cardiology research has found AA access to be a helpful alternative to traditional femoral access when treating patients with peripheral arterial disease and obesity. AA access, as opposed to femoral access for mechanical circulatory support devices, has been shown to increase the range of motion for patients undergoing physical therapy and reduce morbidities associated with prolonged bed rest [22]. These utilizations of alternative access sites further emphasize the growing clinical and surgical value of the AA as research continues to progress. The use of preoperative mapping of the vasculature in the axilla via ultrasound or CT angiogram would be helpful in identifying potential AA variants for surgeries occurring in the axilla and minimizing iatrogenic complications in patients with suspected variants [23].

## Conclusions

Though a bifurcating AA is one of the least common arterial variations found in the axilla, it is a variation that may markedly impact the BP. The most critical implications of axillary neurovascular entrapments, such as those discussed in this case, can significantly affect patients through neuropathic conditions and should be considered clinically, particularly before surgical or radiological interventions. This variation demonstrates the need for physicians to be aware of potential anatomical anomalies and to use preoperative mapping of vasculature in the axilla when possible. Future studies should focus on the prevalence of AA variants, specifically their branching patterns, as a population study to emphasize their rarity and the clinical outcomes of these anomalies.
